# Supplementary material for: FOXP-stabilization of the Il2ra super-enhancer structure augments Treg fitness
Source: bioRxiv. 2026 Apr 17:2026.04.14.718220. Preprint. [Version 1] doi: 10.64898/2026.04.14.718220 (PMC13105002; doi:10.64898/2026.04.14.718220)
Supplement: Supplement 3 [file media-3.pdf]

Table S3. List of publicly available data used in this study, related to Fig. 6, fig. S9 and S10.

File Identity	BioSample	SeqType	Database
SRR5385310	DP	ATAC	DDBJ
SRR5385309	ImCD4SP	ATAC	DDBJ
SRR5385308	CD25+tTreg prec	ATAC	DDBJ
SRR5385307	tTreg	ATAC	DDBJ
SRR5385355	DP, H3K27ac ChIP-Seq	ChIP-seq	DDBJ
SRR5385353	ImCD4SP, H3K27ac ChIP-Seq	ChIP-seq	DDBJ
SRR5385351	CD25+tTreg prec, H3K27ac ChIP-Seq	ChIP-seq	DDBJ
SRR5385349	tTreg, H3K27ac ChIP-Seq	ChIP-seq	DDBJ
GSM7165880	WT Treg-1, H3K27ac ChIP-Seq	ChIP-seq	GEO
GSM7165882	WT Treg-2, H3K27ac ChIP-Seq	ChIP-seq	GEO
GSM7165890	WT Treg, NFAT1 ChIP-Seq	ChIP-seq	GEO
GSM7165888	WT Treg, p300 ChIP-Seq	ChIP-seq	GEO
GSM7213946	Treg, CTCF ChIP-seq	ChIP-seq	GEO
GSM3430729	Foxp1+ Treg, rep1, FOXP1 ChIP-seq	ChIP-seq	GEO
GSM3430735	Foxp1+ Treg, rep1, FOXP3 ChIP-seq	ChIP-seq	GEO
GSM3430737	Foxp1- Treg, rep1, FOXP3 ChIP-seq	ChIP-seq	GEO
GSM2734699	IN1 CD4T +IL2, H3K27Ac ChIP-seq	ChIP-seq	GEO
GSM2734700	IN2 CD4T +IL2, H3K27Ac ChIP-seq	ChIP-seq	GEO
GSM8661318	iTreg, Cont, STAT5B ChIP-seq	ChIP-seq	GEO
GSM8661319	iTreg, IL2, STAT5B ChIP-seq	ChIP-seq	GEO
GSM8661334	ex vivo Treg, STAT5B ChIP-seq	ChIP-seq	GEO
GSM2734684	CD4T, +IL2, STAT5B ChIP-seq	ChIP-seq	GEO
GSM6705657	CD4-positive, alpha-beta thymocyte [Thy]	in situ HiC	GEO
GSM6705655	Treg precursor - CD25+ Foxp3- [Thy]	in situ HiC	GEO
GSM6705675	Treg - thymus - Fox3p-GFP+ [Thy]	in situ HiC	GEO
GSM6705673	"wannabe" Treg - thymus - Foxp3-KO [Thy]	in situ HiC	GEO
GSM6705669	Tcon-SPL	in situ HiC	GEO
GSM6705671	Treg-SPL	in situ HiC	GEO

Table S4. HiSeq pools and adapters for ATAC-seq library preparation, related to Fig. 4.

Pool	*Samples*	*Index*	*Sequence*
**A**	9084	Ad2.4_TCCTGAGC	CAAGCAGAAGACGGCATACGAGATGCTCAGGAGTCTCGTGGGCTCGGAGATGT
	9085	Ad2.6_TAGGCATG	CAAGCAGAAGACGGCATACGAGATCATGCCTAGTCTCGTGGGCTCGGAGATGT
	9086	Ad2.7_CTCTCTAC	CAAGCAGAAGACGGCATACGAGATGTAGAGAGGTCTCGTGGGCTCGGAGATGT
	9106	Ad2.1_TAAGGCGA	CAAGCAGAAGACGGCATACGAGATTCGCCTTAGTCTCGTGGGCTCGGAGATGT
	9186	Ad2.9_GCTACGCT	CAAGCAGAAGACGGCATACGAGATAGCGTAGCGTCTCGTGGGCTCGGAGATGT
	9196	Ad2.11_AAGAGGCA	CAAGCAGAAGACGGCATACGAGATTGCCTCTTGTCTCGTGGGCTCGGAGATGT
	9197	Ad2.12_GTAGAGGA	CAAGCAGAAGACGGCATACGAGATTCCTCTACGTCTCGTGGGCTCGGAGATGT
**B**	9084	Ad2.5_GGACTCCT	CAAGCAGAAGACGGCATACGAGATAGGAGTCCGTCTCGTGGGCTCGGAGATGT
	9107	Ad2.2_CGTACTAG	CAAGCAGAAGACGGCATACGAGATCTAGTACGGTCTCGTGGGCTCGGAGATGT
	9108	Ad2.3_AGGCAGAA	CAAGCAGAAGACGGCATACGAGATTTCTGCCTGTCTCGTGGGCTCGGAGATGT
	9188	Ad2.10_CGAGGCTG	CAAGCAGAAGACGGCATACGAGATCAGCCTCGGTCTCGTGGGCTCGGAGATGT
	9199	Ad2.13_GTCGTGAT	CAAGCAGAAGACGGCATACGAGATATCACGACGTCTCGTGGGCTCGGAGATGT
	9176	Ad2.14_ACCACTGT	CAAGCAGAAGACGGCATACGAGATACAGTGGTGTCTCGTGGGCTCGGAGATGT

Table S5. Key resource.

REAGENT or RESOURCE	SOURCE	IDENTIFIER
**Antibody**		
BD Pharmingen™ APC Rat Anti-Mouse CD62L (MEL-14)	BD biosciences	553152
BD Pharmingen™ Alexa Fluor® 647 Mouse anti-BrdU (3D4)	BD biosciences	560209
BD Horizon™ BUV395 Rat Anti-Mouse CD25 (PC61)	BD biosciences	564022
BD Horizon™ BUV496 Hamster Anti-Mouse CD3ε (145-2C11)	BD Biosciences	612955
Brilliant Violet 785™ anti-mouse CD3 (17A2)	Biolegend	100232
PE anti-mouse CD4 (GK1.5)	Biolegend	100408
Alexa Fluor® 700 anti-mouse CD4 (GK1.5)	Biolegend	100430
Pacific Blue™ anti-mouse CD5 (53-7.3)	Biolegend	100641
PerCP/Cyanine5.5 anti-mouse CD8a (53-6.7)	Biolegend	100734
PE/Cyanine7 anti-mouse CD25 (3C7)	Biolegend	101916
PE anti-mouse CD25 (PC61)	Biolegend	102008
Ultra-LEAF™ Purified anti-mouse CD28 (37.51)	Biolegend	102116
Pacific Blue™ anti-mouse/human CD44 (IM7)	Biolegend	103020
Pacific Blue™ anti-mouse/human CD45R/B220 (RA3-6B2)	Biolegend	103227
Pacific Blue™ anti-mouse CD24 Antibody (M1/69)	Biolegend	101819
Brilliant Violet 711™ anti-mouse CD69 (H1.2F3)	Biolegend	104537
PE/Cyanine7 anti-mouse CD152 (UC10-4B9)	Biolegend	106314
APC/Cyanine7 anti-mouse TCR β chain (H57-597)	Biolegend	109220
Alexa Fluor® 594 anti-mouse CD45.2 (104)	Biolegend	109850
PerCP/Cyanine5.5 anti-mouse CD45.1 (A20)	Biolegend	110727
Brilliant Violet 421™ anti-mouse FOXP3 (MF-14)	Biolegend	126419
PE/Dazzle™ 594 anti-mouse/human Helios (22F6)	Biolegend	137232
PerCP/Cyanine5.5 anti-mouse CD304 (3E12)	Biolegend	145208
Alexa Fluor® 488 anti-GFP (FM264G)	Biolegend	338008
PE/Dazzle™ 594 anti-mouse Ki-67 (16A8)	Biolegend	652427
Brilliant Violet 421™ anti-mouse CD122 (IL-2Rβ) (5H4)	Biolegend	105919
PE anti-mouse CD122 (IL-2Rβ) Antibody (TM-β1)	Biolegend	123210
PE anti-mouse Qa-2 Antibody (695H1-9-9)	Biolegend	121715
Brilliant Violet 650™ anti-rat CD90/mouse CD90.1 (Thy-1.1) (OX-7)	Biolegend	202533

PE/Cyanine7 anti-mouse CD132 (common γ chain) (TUGm2)	Biolegend	132311
Brilliant Violet 785™ anti-mouse CD45.2 (104)	Biolegend	109839
Brilliant Violet 421™ anti-mouse CD90.2 (Thy-1.2) (53-2.1)	Biolegend	140327
PE anti-mouse CD45.1 (A20)	Biolegend	110708
PerCP anti-mouse CD45 (30-F11)	Biolegend	103130
PerCP anti-mouse IL-17A (TC11-18H10)	Biolegend	506944
PerCP/Fire™ 780 anti-mouse CD183 (S18001A)	Biolegend	155926
APC/Cyanine7 anti-mouse CD62L (MEL-14)	Biolegend	104428
Purified anti-mouse CD25 Antibody (3C7)	Biolegend	101902
InVivoMAb anti-mouse CD16/CD32 (2.4G2)	BioXCell	BE0307
InVivoMAb anti-mouse CD8α (2.43)	BioXCell	BE0061
InVivoMAb anti-mouse MHC Class II (I-A/I-E) (M5/114)	BioXCell	BE0108
InVivoMAb anti-mouse CD3ε (145-2C11)	BioXCell	BE0001-1
PE anti-mouse FoxP1 (D35D10)	Cell Signaling Technology	99657
eBioscience™ APC anti-mouse FOXP3 (FJK-16s)	Invitrogen	17-5773-82
eBioscience™ PE anti-Phospho-STAT5 (Tyr694) Monoclonal Antibody (SRBCZX)	Invitrogen	12-9010-42e
BioMag Goat Anti-Rat IgG	Qiagen	310107
Histone H3K27ac (pAb)	Active Motif	39134
**Chemical, Reagents**		
RBC Lysis Buffer (10X)	Biolegend	420302
10% Tween-20	BioRad	1662404
Percoll® PLUS	Cytiva	GE17-5445-02
GemCell FBS	GeminiBio	100-500
1X PBS pH 7.2	Gibco	20012050
HEPES (1 M)	Gibco	15630106
Sodium Pyruvate (100 mM)	Gibco	11360070
PBS (10X), pH 7.4	Gibco	70011069
GlutaMAX™ Supplement	Gibco	35050061
RPMI 1640 Medium, no glutamine	Gibco	21870076
RPMI 1640 Medium, no phenol red	Gibco	11835030
Digitonin (5%)	Invitrogen	BN2006
Normal Rat Serum	Invitrogen	01-9601
Proteinase K Solution	Invitrogen	4333793
Streptavidin	Invitrogen	434302

UltraPure™ 0.5M EDTA, pH 8.0	Invitrogen	15575020
Brefeldin A	Invitrogen	B7450
BrdU (5-Bromo-2'-Deoxyuridine)	Invitrogen	B23151
Recombinant Human TGF-β1 (CHO derived)	PeproTech	100-21C
Recombinant Mouse IL-2 Protein	R&D Systems	402-ML
DNase I recombinant, RNase-free	Roche	4716728001
Liberase™ TL Research Grade	Roche	5401020001
Liberase™ TM Research Grade	Roche	5401119001
Protector RNase Inhibitor (10000 UNITS)	Roche	3335402001
Penicillin-Streptomycin	Sigma-Aldrich	P4333
(2-Hydroxypropyl)-β-cyclodextrin solution	Sigma-Aldrich	H5784
Bovine Serum Albumin	Sigma-Aldrich	A2153
Dimethyl Sulfoxide	Sigma-Aldrich	D8418
Dulbecco's Modified Eagle's Medium - high glucose	Sigma-Aldrich	D5796
Ionomycin calcium salt	Sigma-Aldrich	I0634
Phorbol 12-myristate 13-acetate	Sigma-Aldrich	P8139
Tamoxifen	Sigma-Aldrich	T5648
Paraformaldehyde, 16% w/v	Alfa Aesar	43368
2-Mercaptoethanal	Sigma-Aldrich	63689
**Commercial assays**		
D1000 Ladder	Agilent	5067-5586
D1000 Reagents	Agilent	5067-5583
D1000 ScreenTape	Agilent	5067-5582
TaqMan™ Universal Master Mix II, with UNG	Applied Biosystems	4440042
BD Pharmingen™ APC BrDU Kit	BD biosciences	552598
SPRIselect, 5 mL	Beckman Coulter	B23317
Zombie Aqua™ Fixable Viability Kit	Biolegend	423102
Zombie NIR™ Fixable Viability Kit	Biolegend	423105
Dynabeads™ Mouse T-Activator CD3/CD28 for T-Cell Expansion and Activation	Gibco	11456D
CellTrace™ Violet Cell Proliferation Kit, for flow cytometry	Invitrogen	C34557
CountBright™ Absolute Counting Beads	Invitrogen	C36950
eBioscience™ Annexin V Apoptosis Detection Kits PE	Invitrogen	88-8102-72
eBioscience™ Foxp3 / Transcription Factor Fixation/Permeabilization Concentrate and Diluent	Invitrogen	00-5521-00
eBioscience™ Permeabilization Buffer (10X)	Invitrogen	00-8333-56

GFP BrightComp eBeads™ Compensation Bead Kit	Invitrogen	A10514
CellEvent™ Caspase-3/7 Detection Reagents, Red, Powder	Invitrogen	C10430
UltraComp eBeads™ Compensation Beads	Invitrogen	01-2222-42
SuperScript™ III First-Strand Synthesis System	Invitrogen	18080051
ArC™ Amine Reactive Compensation Bead Kit	Invitrogen	A10346
NEBNext® High-Fidelity 2X PCR Master Mix	NewEngland Biolab	M0541S
MinElute PCR Purification Kit (50)	Qiagen	28004
RNeasy Micro Kit (50)	Qiagen	74004
**TaqMan probes**		
Actb	Thermo Fisher	Mm02619580_g1
Foxp1	Thermo Fisher	Mm01181991_g1
Foxp2	Thermo Fisher	Mm00475030_m1
Foxp3	Thermo Fisher	Mm00475162_m1
Foxp4	Thermo Fisher	Mm01269228_g1
Il2ra	Thermo Fisher	Mm01340213_m1
**Animals**		
$Foxp3^{tm9(EGFP/cre/ERT2)Ayr}/J$	Jackson Laboratory	016961
$Foxp1^{fl/fl}$	from H. Tucker	Feng et al.(*9*)
$Foxp4^{fl/fl}$	from E. Morrissey	Li et al.(*42*)
$Foxp3^{YFP/cre}$	from A. Rudensky	Rubstov et al.(*43*)
$Cd4^{Cre/ERT2}$	from F. Gounari	Aghajani et al.(*52*)
$B6.129X1\text{-}Gt(ROSA)26Sor^{tm1(EYFP)Cos}/J$	from F. Costantini	Srinivas et al. (*44*)
$B6.PL\text{-}Thy1^{a}/CyJ$	Jackson Laboratory	000406
B6.SJL-Ptprca Pepcb/BoyJ	Jackson Laboratory	002014

B6.129P2-Tcrb^{tm1Mom} Tcrd^{tm1Mom}/J	Jackson Laboratory	002122
**Software and algorithm**		
Flow Jo™ (Ver10.10)	BD Biosciences	https://www.flowjo.com
GraphPad Prism (Ver10.2.2)	GraphPad Software	https://www.graphpad.com/
R (Ver4.5.0)	R Foundation	https://www.r-project.org
RStudio	Posit Software	https://posit.co
Docker	Docker Inc.	https://www.docker.com
IGV	UC San Diego	https://igv.org
