# Supplementary material for: FOXP-stabilization of the Il2ra super-enhancer structure augments Treg fitness
Source: bioRxiv. 2026 Apr 17:2026.04.14.718220. Preprint. [Version 1] doi: 10.64898/2026.04.14.718220 (PMC13105002; doi:10.64898/2026.04.14.718220)
Supplement: 4 [file NIHPP2026.04.14.718220v1-supplement-4.pdf]

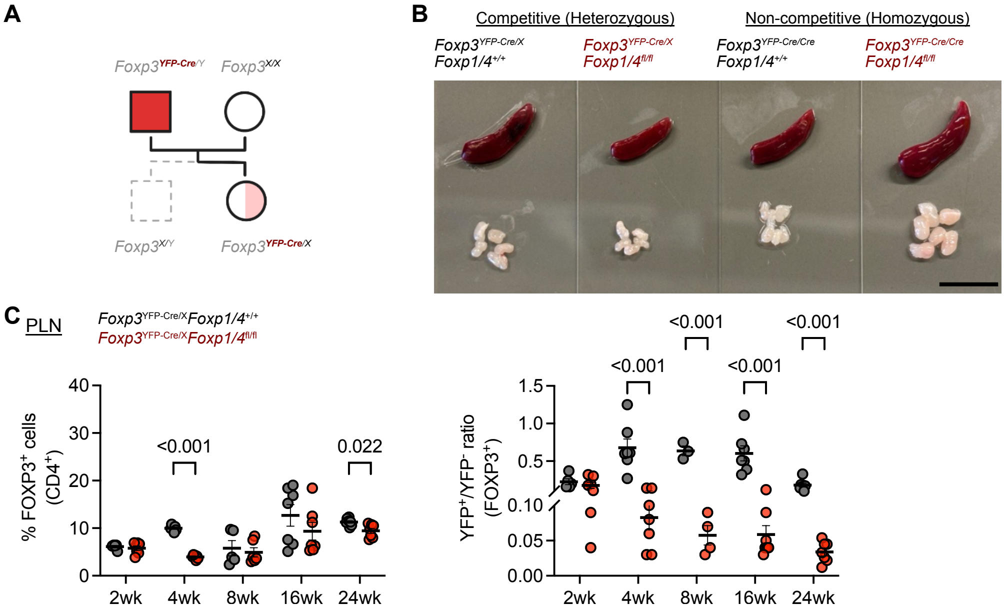

fig. S1. Breeding and phenotypic assessment of *Foxp3$^{YFP\text{-}Cre/X}$Foxp1/4$^{fl/fl}$* mice.

(A) Breeding strategy of generating *Foxp3$^{YFP\text{-}Cre/X}$Foxp1/4$^{fl/fl}$* female heterozygotes. (B) A representative appearance of spleen and peripheral lymph nodes from *Foxp3$^{YFP\text{-}Cre/X}$Foxp1/4$^{fl/fl}$* heterozygotes and *Foxp3$^{YFP\text{-}Cre/Cre}$Foxp1/4$^{fl/fl}$* homozygous at 8 weeks of age. (Scale bar = 1cm). (C**)** The frequency of FOXP3$^+$ Treg within CD4$^+$ cell and the YFP$^+$/YFP$^-$ ratio at the indicated time points (n= 5 to 8 per group). Data are presented as mean ± SEM, each dot represents data from an individual mouse. Statistical analysis: two-tailed unpaired t-test, *P* values are shown for significant difference ($P < 0.05$).

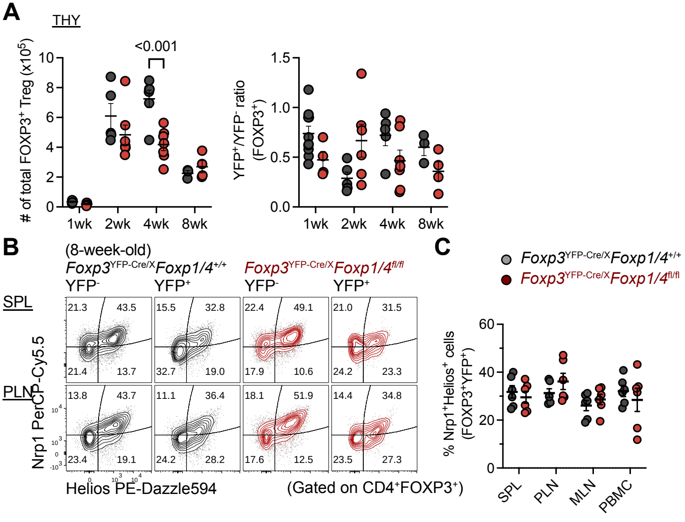

fig. S2. Loss of FOXP1/FOXP4 does not affect tTreg development or peripheral seeding.
(A) Total number and YFP⁺/YFP⁻ ratio of Tregs in thymus of mice at 1, 2, 4 and 8 weeks of age (n = 4-10 per group). (B and C) Representative flow cytometry plots and quantitation illustrate the proportion of the tTreg (Nrp1⁺Helios⁺) compartment in the splenic lymphoid organs (SLOs) of 8-week-old mice. Statistical analysis: two-tailed unpaired t-test, *P* values are shown for significant difference ($P < 0.05$).

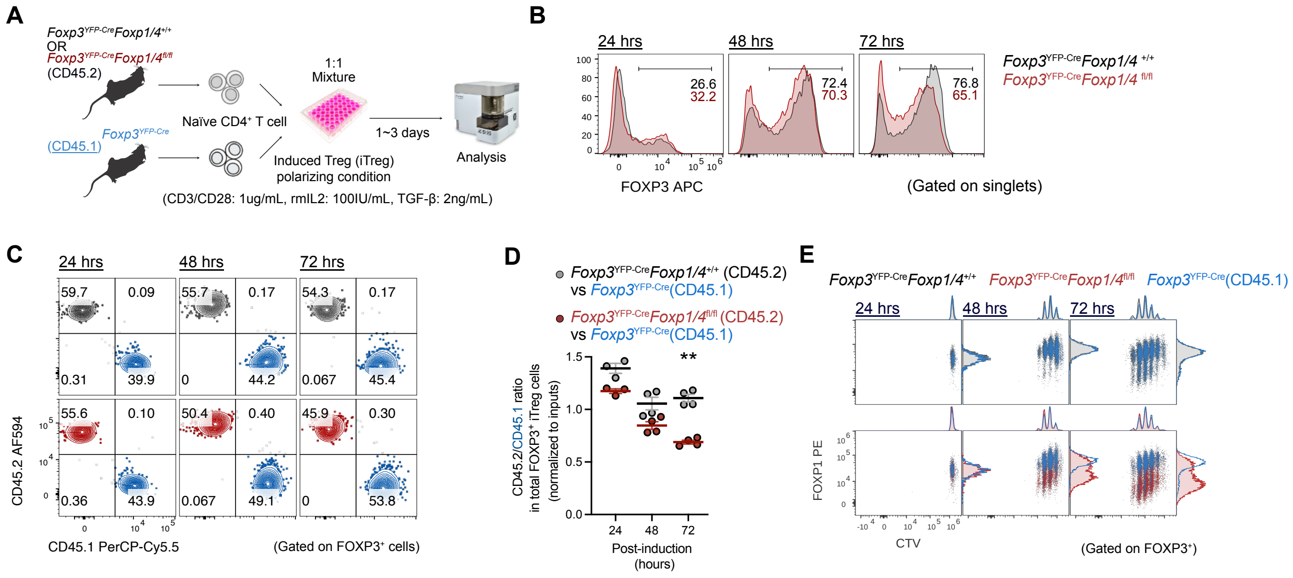

fig. S3. FOXP1/FOXP4-deficient iTreg develop and proliferate normally.

(A) Schematic workflow for studying *in vitro* iTreg polarization under competitive condition. (B) Proportion of newly generated FOXP3+ iTreg cells (boxed quadrant) during the first 24 and 48 hours of polarization. (C) Representative flow cytometric plots of iTreg competitiveness after induction, and (D) quantification of their relative ratio within FOXP3+ cells (n=3 to 4 per group, representative of two independent experiments). (E) CTV-labeled tracking of cell proliferation was measured at 24-hour intervals during competitive co-culture iTreg polarization. Staked histogram showing FOXP1 expression level and cell proliferation during iTreg induction. Statistical analysis: two-tailed unpaired t-test, **= P <0.01.

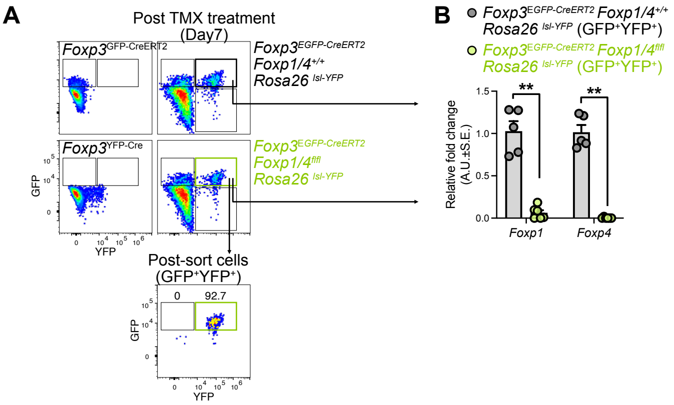

fig. S4. Acute depletion of FOXP1 and FOXP4 in Tregs.
(A) Representative flow cytometric plots of sorted YFP$^+$ cells from GFP$^+$ Tregs. (B) Quantification of relative *Foxp1* and *Foxp4* transcripts in sorted YFP$^+$GFP$^+$ cells seven days post-inducible knockout. Data are presented as mean ± SEM, each dot represents data from an individual mouse. Statistical analysis: two-tailed unpaired t-test, ** = *P* <0.01

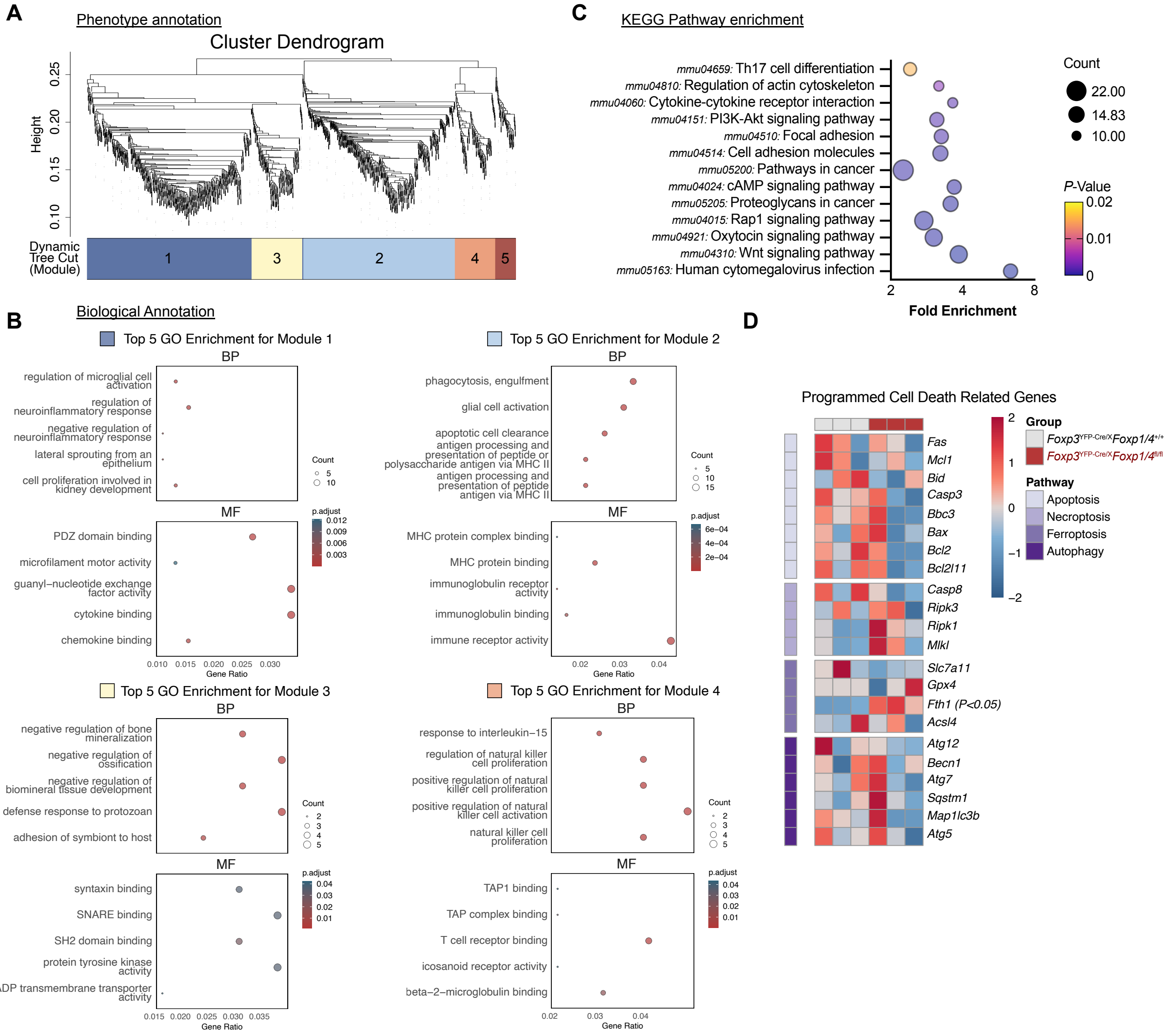

fig. S5. Transcriptomic annotation of FOXP1/FOXP4 deficient Tregs.
(A) Dendrogram of DEGs from RNA-seq data clustered by weighted gene co-expression network analysis, using hierarchical clustering (McQuitty method) and dynamic tree cutting based on TOM dissimilarity. (B) Biological function annotation (Biological Process; BP and Molecular Function; MF) of the top 4 clustered modules identified in (A). (C) KEGG pathway enrichment analysis of genes shared between RNA-seq and ATAC-seq datasets. (D) Heatmap of transcription level of programmed cell death-related genes.

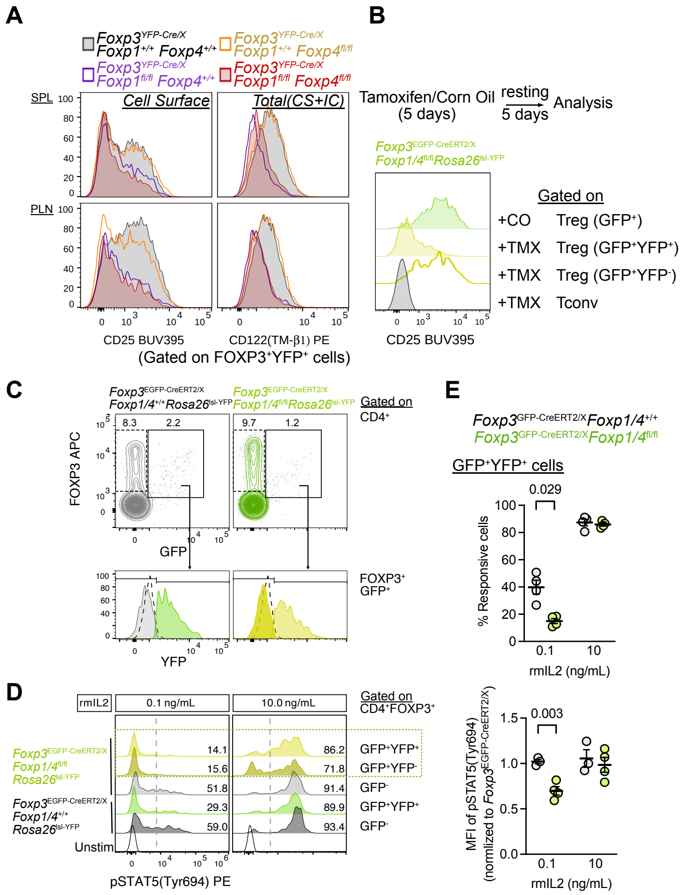

fig. S6. FOXP1 is required for competition for IL-2 responsiveness and is associated with CD25.

(A) Histogram of cell surface CD25 and total CD122 (cell surface plus intracellular; CS+IC) expression level in FOXP3⁺YFP⁺ cells from all four strains. (B) Stacked histogram of cell surface CD25 in *Foxp3*-driven FOXP1/FOXP4 acutely depleted Treg cells. (C and D) Representative flow cytometric plots of gating strategy (C) and pSTAT5$_{Tyr694}$ levels in PLN Tregs obtained from FOXP1/FOXP4 acutely depleted mice (D). (E) Quantification of the proportion of IL-2-responsive cells and relative pSTAT5$_{Tyr694}$ levels in Tregs from (D) (n = 4 per group). Data are presented as mean ± SEM, each dot represents data from an individual mouse. Statistical analysis: two-tailed unpaired t-test, exact *P* values are shown for significant difference ($P < 0.05$).

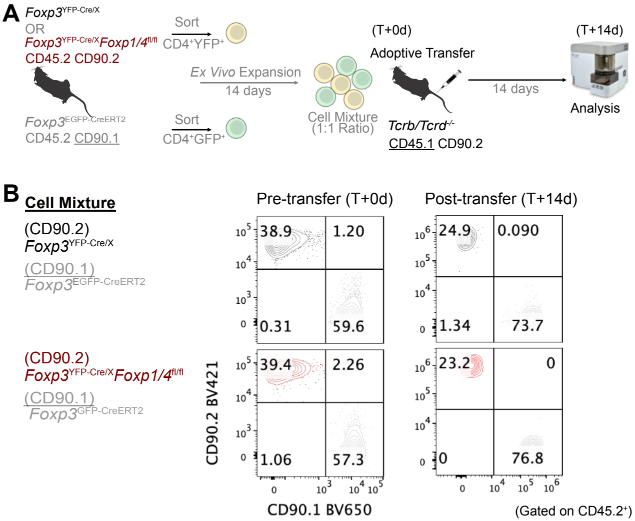

fig. S7. No competitive disadvantage of FOXP1/FOXP4-deficient Tregs in the absence of conventional T cells.

(A) Schematic of workflow to assess Treg competitiveness for IL-2 *in vivo* by using *ex vivo* expanded Tregs. (B) Representative flow cytometric plots showing the relative competitiveness of adoptively transferred donor cells in congenic *Tcrb/Tcrd*$^{-/-}$ hosts.

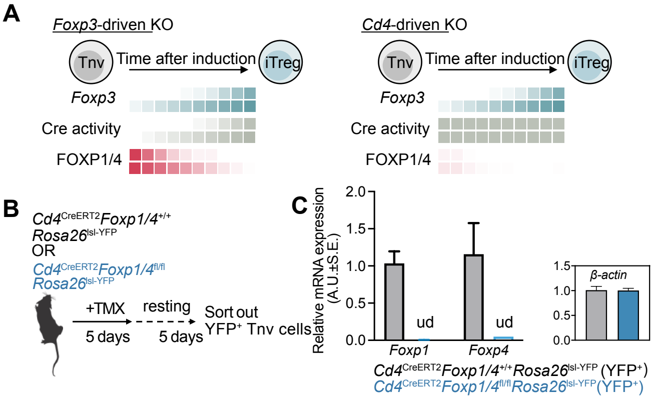

fig. S8. CD4$^{Cre\text{-}ERT2}$-mediated deletion of *Foxp1/4.*

(A) Cartoon illustration showing how FOXP1/FOXP4 levels change during iTreg polarization under the control of *Foxp3*-driven or *Cd4*-driven Cre activity. (B) Workflow of obtaining *Foxp1/4*-deficient Tnv from *Cd4*^{CreERT2} mice. (C) Quantification of *Foxp1* and *Foxp4* knockout efficiency in *Cd4*-driven inducible knockout models.

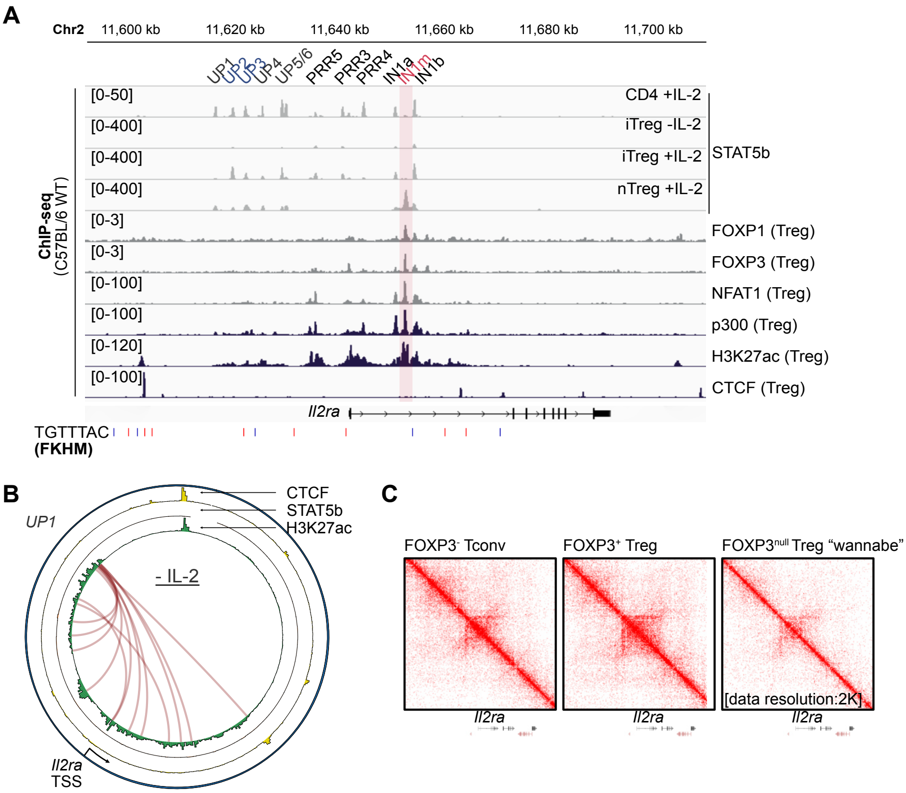

fig. S9. Regulatory landscape and chromatin architecture of Il2ra locus in Treg.
(A) STAT5b ChIP-seq tracks at the *Il2ra* locus during Treg development, and ChIP-seq of FOXP1, FOXP3, NFAT1, P300, H3K27ac, and CTCF at the *Il2ra* locus in wildtype mature Tregs. Reanalyzed from publicly accessible datasets (Supplementary Table 3). Red shading represents the IN1m region of the *Il2ra* locus. (B) RNA-PolII ChIA-PET plot of chromatin interactions in the *Il2ra* locus of wild-type CD4 cells, without IL-2 stimulation. (C) In-situ Hi-C plots at the *Il2ra* locus in Tconv, Treg from spleen and Treg "wannabe" (samples GSM6705669, GSM6705671, and GSM6705673).

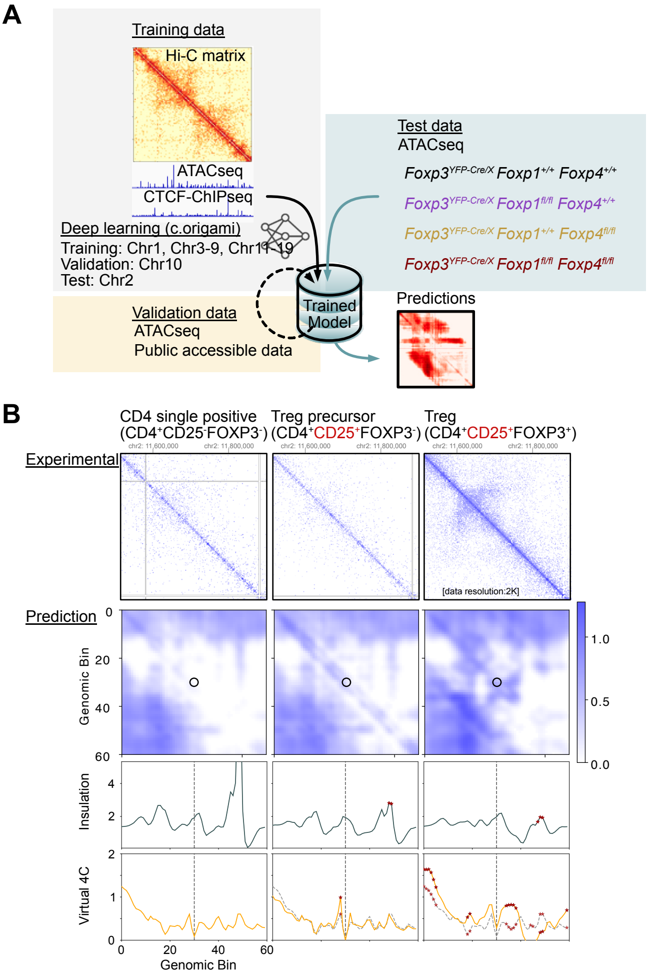

fig. S10. Treg-specific trained model for *in silico* Hi-C analysis.
(A) Schematic workflow for *in silico* Hi-C prediction in mouse Tregs using publicly accessible data (Hi-C:GSM6705675, CFCF-ChIP: GSM7213946, ATAC-seq: GSM9230397). (B) Validation of the predictive performance of the trained model. Experimental data were obtained from in situ Hi-C of thymic Tregs, the prediction matrix was generated based on analysis of ATAC-seq from thymic Tregs (SRR5385309, SRR5385308, and SRR5385307). Insulation score and virtual 4C profiles calculated to predict TAD formation at *Il2ra* locus. Open circle on the matrix and the vertical line on both the insulation score and virtual 4C indicates the *Il2ra* TSS.
